# Collapse of the tropical and subtropical North Atlantic CO_2_ sink in boreal spring of 2010

**DOI:** 10.1038/srep41694

**Published:** 2017-01-30

**Authors:** J. Severino P. Ibánhez, Manuel Flores, Nathalie Lefèvre

**Affiliations:** 1 Department of Oceanography – DOCEAN, Federal University of Pernambuco – UFPE, Av. Arquitetura, s/n, Cidade Universitária, 50740-550, Recife-PE, Brazil; 2 IRD-LOCEAN, Sorbonne Universités (Université Pierre et Marie Curie-CNRS-MNHN), 4 place Jussieu, 75252 Paris Cedex 05, France

## Abstract

Following the 2009 Pacific El Niño, a warm event developed in the tropical and subtropical North Atlantic during boreal spring of 2010 promoted a significant increase in the CO_2_ fugacity of surface waters. This, together with the relaxation of the prevailing wind fields, resulted in the reversal of the atmospheric CO_2_ absorption capacity of the tropical and subtropical North Atlantic. In the region 0–30°N, 62–10°W, this climatic event led to the reversal of the climatological CO_2_ sink of −29.3 Tg C to a source of CO_2_ to the atmosphere of 1.6 Tg C from February to May. The highest impact of this event is verified in the region of the North Equatorial Current, where the climatological CO_2_ uptake of −22.4 Tg for that period ceased during 2010 (1.2 Tg C). This estimate is higher than current assessments of the multidecadal variability of the sea-air CO_2_ exchange for the entire North Atlantic (20 Tg year^−1^), and highlights the potential impact of the increasing occurrence of extreme climate events over the oceanic CO_2_ sink and atmospheric CO_2_ composition.

Anthropogenic CO_2_ emission to the atmosphere is widely considered the main cause of current climate change. Since the industrial revolution, the oceans have absorbed about 40–50% of all the anthropogenic CO_2_ emissions^1^,^2^, thus mitigating its effects over the Earth climate system. Nevertheless, studies have suggested that the oceanic C sink may be decreasing for the last 50 years^3^,^4^. Whether these changes are caused from anthropogenic climate change or internal climate variability is still uncertain^4^,^5^,^6^, but they could significantly impact future atmospheric CO_2_ levels.

The North Atlantic north of 18°N is one of the oceanic regions of strongest CO_2_ uptake (420 ± 110 Tg C y^−1^) representing 30% of the global oceanic CO_2_ sink^7^, and an estimated interannual and multidecadal CO_2_ uptake variability of 20 Tg C yr^−1 ^7,8,9. The area of the North Atlantic with CO_2_ uptake that is most sensitive to climate forcing (changes in sea surface temperature (SST) and wind speed) is the subtropical North Atlantic^10^. There, the sea-air CO_2_ exchange is mainly controlled by sea surface temperature (SST) changes due to its permanent oligotrophic conditions outside upwelling areas, thus presenting the strongest seasonal variability in the sea-air CO_2_ exchange of this ocean^10^. Recent warming identified in the region, partially linked to anthropogenic forcing, is already reducing its CO_2_ uptake^11^.

Large-scale climate modes such as the North Atlantic Oscillation (NAO), the Atlantic Multidecadal Oscillation (AMO) and the El Niño-Southern Oscillation (ENSO) can mitigate or exacerbate anthropogenic-driven SST increase in the North Atlantic and its effects over the sea-air CO_2_ exchange^12^. In 2009, a strong El Niño event occurred in the Pacific. ENSO events are known to promote positive SST anomalies in the northern tropical Atlantic through a teleconnection driven through the troposphere with a time lag of a few months^13^. In boreal spring of 2010, this event coincided with a strong positive AMO resulting in a strong positive SST anomaly associated with negative wind speed anomalies in the tropical Atlantic^14^,^15^(Fig. 1). Its impact in the equatorial Atlantic sea-air CO_2_ exchange has been explored by Lefèvre *et al*.^14^, who showed that during this period the Intertropical Convergence Zone (ITCZ) was shifted northward compared to its climatological position, associated with a significant reduction of rainfall. The CO_2_ undersaturation promoted by the intense rainfall associated with the ITCZ was thus significantly reduced and, consequently, CO_2_ outgassing in this area increased. As their study mainly focused on the Western equatorial Atlantic, the impact of this climatic event in the sea-air CO_2_ exchange in the North Atlantic is currently unknown.

We have analyzed synoptic underway data collected onboard two Voluntary Observing Ships (VOS) that sailed across the North Atlantic during boreal spring 2010 (Monte Olivia/Rio Blanco sailing the route France-Brazil and Colibri sailing the route France-French Guyana; see Fig. 2 upper panels and Supplementary Table S1 for details on the route of the vessels). We have also used the data collected onboard these VOS during boreal spring of other years for comparison. The aim of this study is to identify the extension and quantify the impact of the SST anomaly observed in the North Atlantic climatic event of boreal spring 2010 (Fig. 1) with regard to the CO_2_ uptake capacity of the basin.

## Results and Discussion

### Latitudinal distribution of sea surface temperature, salinity and the fugacity of CO_2_

Underway SST measured during 2010 in the North Atlantic is significantly higher than that measured in other years along both the Monte Olivia/Rio Blanco and the Colibri tracks (differences up to 3.4 °C during March; Fig. 2.2). These SST differences are registered from ~30°N to the equator along the Monte Olivia/Rio Blanco tracks and to ~10°N along the route of the Colibri. The higher underway SST measured during 2010 is accompanied by a significant increase in the measured sea surface CO_2_ fugacity (fCO_2sw_). The thermodynamic effect of SST changes on fCO_2sw_ results in ~4% fCO_2sw_ increase for every °C due to the reduction of CO_2_ solubility in seawater at increasing temperatures^16^. This thermodynamic effect of temperature on fCO_2sw_ is well exemplified in the fCO_2sw_ data from 2010.

Latitudinal sea surface salinity (SSS) differences among years are associated with the main freshening sources to the basin, i.e. the Amazon River plume (in the southernmost part of the Colibri tracks)^17^ and the ITCZ (in the Southern Hemisphere along the Monte Olivia/Rio Blanco tracks)^14^. These freshening sources to the basin promote a lowering of fCO_2sw_ and thus explain the fCO_2sw_ variability in these regions (e.g. Fig. 2.14). Nevertheless, the mechanisms by which these two freshening sources affect the measured fCO_2sw_ are different. The main process explaining the lowering in the fCO_2sw_ in the Amazon plume compared to the surrounding oceanic waters is the C drawdown caused by the intense primary production associated with its waters^17^. In the case of the effect caused by the intense rainfall associated with the ITCZ, chemical dilution of total alkalinity (TAlk) and dissolved inorganic C (DIC) and atmospheric DIC deposition are the main effects over the carbonate system of the oceanic surface waters. Since rainfall commonly contains zero TAlk and near zero DIC, the overall effect on fCO_2sw_ is to lower its values^18^.

We also observe latitudinal SSS gradients during February and March 2009 at the ~24–18°N latitudinal band along the Monte Olivia/Rio Blanco tracks, concomitant with rapid changing latitudinal SST and fCO_2sw_ (Fig. 2). In these two cruises, measured fCO_2sw_ was similar of even higher than that registered during 2010 despite the lower SST. This area, locus of the Canary Current (CC), presents a complex system of mesoscale features such as eddies and fronts and is locus of one of the four main Eastern Boundary Upwelling Systems^19^. The sharp latitudinal SST, SSS and fCO_2sw_ gradients present in these two cruises (February and March 2009) may be associated with upwelling waters and mesoscale transport variability in the area which would explain the high fCO_2sw_ values registered in this region.

The different water masses transported by the surface current system (Fig. 3) are also clearly reflected in the measured fCO_2sw_. The equatorial region is dominated by the South Equatorial Current (SEC) that transports Southern Hemisphere CO_2_-rich waters and the transition between waters originated from both Hemispheres is represented by the different fCO_2sw_ along the Monte Olivia/Rio Blanco tracks at about 4°N (Fig. 1.13 to 1.18).

### Influence of sea surface circulation on SST and sea-air CO_2_ exchange

Using the Ocean Surface Current Analyses – Real time (OSCAR) data, we estimate the location of the surface currents present along the tracks of the VOS used here (Supplementary Figure S1). From North to South, the large-scale surface currents crossed by the Monte Olivia/Rio Blanco are the CC, the North Equatorial Current (NEC), the North Equatorial Counter Current (NECC) and two branches of the SEC (hereby termed North and South SEC; nSEC and sSEC; Fig. 2 upper panels and 3). Further West, after crossing the subduction region of the North Atlantic subtropical gyre, the Colibri crosses the NEC, the NECC and the North Brazil Current (NBC; Fig. 2 upper panels and 3)^20^.

Excluding the data from the cruises of the Monte Olivia/Rio Blanco apparently affected by upwelling waters in the CC region, we observe significantly higher SST anomalies during 2010 compared to the other years (p < 0.05; Supplementary Figure S1) in all the areas delimited by the surface currents but the subduction region of the subtropical gyre (crossed by the Colibri) and the sSEC (crossed by the Monte Olivia/Rio Blanco). There, SST anomalies remain always below 1 °C. Similarly, ΔfCO_2_ (the difference between fCO_2sw_ and the atmospheric fCO_2_, fCO_2atm_; positive values denote sea CO_2_ outgassing) is systematically higher during 2010 than during the other years (p < 0.0001; Fig. 4, panels labeled a), except in the subtropical gyre. The highest ΔfCO_2_ differences between the cruises from 2010 and those from other years are found in the NEC (18.2 ± 0.8, 33.1 ± 0.8, 6.2 ± 0.4 and 7.8 ± 1.2 μatm from February to May; p < 0.0001), except in May along the Monte Olivia/Rio Blanco track (−0.4 ± 0.6 μatm). The negative ΔfCO_2_ characteristic of this current during boreal spring is significantly reduced during 2010, with values close to zero and even reversal of the direction of the sea-air CO_2_ exchange during March (ΔfCO_2_ 3.9 ± 0.2 μatm, n = 2672, p < 0.0001; Fig. 4.2.a). In the region of the sSEC, ΔfCO_2_ during February, March and May 2010 is also significantly higher than those registered during the other years included in this study (p < 0.0001; Fig. 4.1.a, 4.2.a and 4.6.a). The abnormal position and intensity of the ITCZ during 2010 explains the increased ΔfCO_2_ observed in this area^14^.

Despite the close longitudinal proximity of the tracks of the two VOS used here in the North of the study area, the significant positive SST anomalies observed in the CC (crossed by the Monte Olivia/Rio Blanco) are not observed in the subduction region of the North Atlantic subtropical gyre (crossed by the Colibri). The CC flows to the South and therefore is not a significant water transport pathway to the subduction region of the subtropical gyre. Furthermore, the sSEC, which transports Southern Hemisphere waters, shows weak SST anomalies during the studied period. At the basin scale, the positive SST anomalies registered from February to May 2010 seem to be generated in the eastern tropical Atlantic, then spreading to west and south following the surface current system. Thus, advection by the surface current system seems to contribute to the spatial distribution of the SST anomalies and ΔfCO_2_ increase observed in 2010, mainly restricted to the tropical and subtropical North Atlantic.

### Physical contributors to the anomalous sea-air CO_2_ exchange during 2010

To evaluate the potential contribution of physical parameters to the sea-air CO_2_ flux anomaly of 2010, we compare the underway sea-air CO_2_ flux to that normalized to monthly SST, SSS, wind intensity, fCO_2atm_ and fCO_2sw_. Excluding the data apparently affected by upwelling waters in the CC and SSS < 35 (thus excluding the Amazon plume), the normalized CO_2_ flux during 2010 is never significantly higher than that calculated for the other cruises in the area of the CC, the NEC and the NECC (p > 0.05; Supplementary Figure S2). In the region of the nSEC and sSEC, the normalized CO_2_ flux during 2010 is still slightly higher than during the remaining cruises (unless during April in the sSEC), which may be related to the uncertainties of the thermodynamic effect of rainfall (ITCZ) over fCO_2sw_^21^. These results suggest that in the tropical and subtropical North Atlantic, the parameters not accounted in the CO_2_ flux normalization (i.e. primary production, fCO_2sw_ increase due to fCO_2atm_ increase) had little contribution to the CO_2_ flux anomaly observed in 2010.

**Figure 1:**
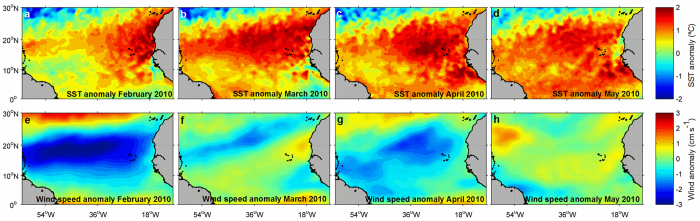
Basin-scale SST (**a** to **d**) and wind speed (**e** to **h**) anomalies during February to May 2010. SST and wind speed anomalies were calculated as the deviation of the monthly data from the climatological SST and wind speed calculated for the period 1994–2013. Monthly SST was calculated from the daily NOAA optimum interpolation (OI) SST V2 data (0.25° resolution; NOAA/OAR/ESRL PSD, Boulder, Colorado, USA; http://www.esrl.noaa.gov/psd/). Wind speed was obtained from the European Centre for Medium-Range Weather Forecasts (ECMWF) monthly reanalysis data set (ERA-interim, 0.25° resolution). The maps were generated with Matlab 2016a, the M_Map 1.7 toolbox (https://www.eoas.ubc.ca/~rich/map.html) and using the ETOPO2 Global 2 Arc-minute Ocean Depth and Land Elevation (National Geophysical Data Center/NESDIS/NOAA/U.S. Department of Commerce. 2001. ETOPO2, Global 2 Arc-minute Ocean Depth and Land Elevation from the US National Geophysical Data Center (NGDC). Research Data Archive at the National Center for Atmospheric Research, Computational and Information Systems Laboratory. http://dx.doi.org/10.5065/D6668B75. Accessed 1/03/2015).

**Figure 2:**
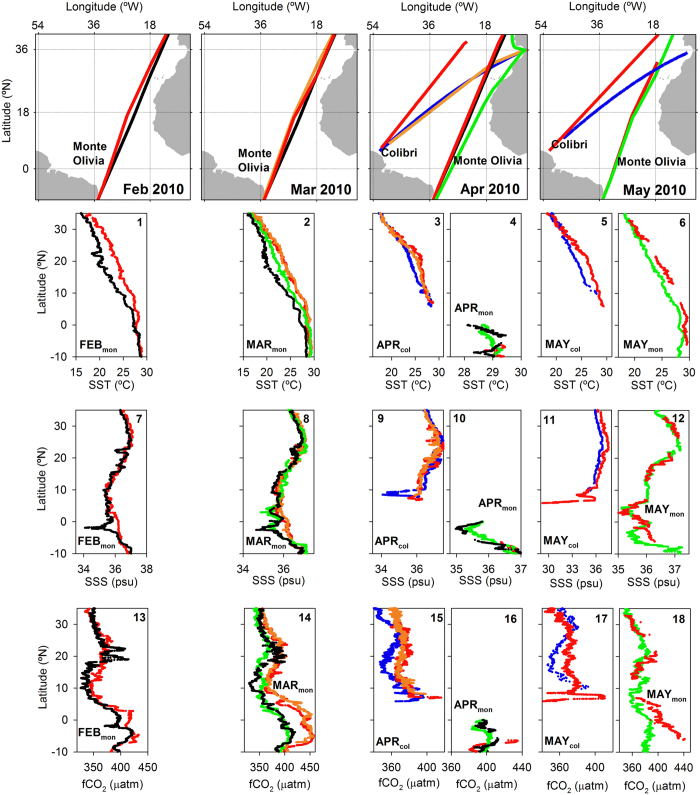
Underway measurements performed onboard the Monte Olivia/Rio Blanco/Colibri used in this study. Upper panels show the route followed by each cruise across the North Atlantic. The latitudinal distribution of underway SST (panels 1–6), SSS (panels 7–12) and fCO_2sw_ (panels 13–18) for the voyages used here are shown. Color code denotes the year of each cruise: blue represents 2007, black 2009, red and orange 2010, and green 2011. Subscript “mon” denotes data collected onboard the Monte Olivia/Rio Blanco, while subscript “col” refers to the Colibri. Note that the route of the Monte Olivia/Rio Blanco during March 2010 and 2011 overlap. The maps were generated with Matlab 2016a, the M_Map 1.7 toolbox (https://www.eoas.ubc.ca/~rich/map.html) and using the ETOPO2 Global 2 Arc-minute Ocean Depth and Land Elevation (National Geophysical Data Center/NESDIS/NOAA/U.S. Department of Commerce. 2001. ETOPO2, Global 2 Arc-minute Ocean Depth and Land Elevation from the US National Geophysical Data Center (NGDC). Research Data Archive at the National Center for Atmospheric Research, Computational and Information Systems Laboratory. http://dx.doi.org/10.5065/D6668B75. Accessed 1/03/2015).

**Figure 3:**
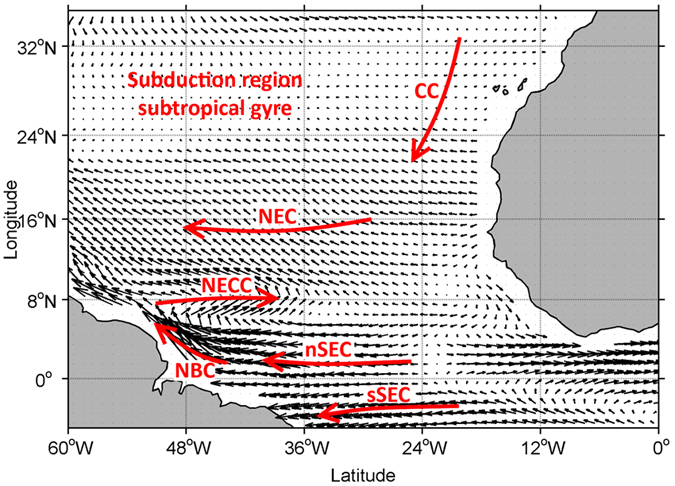
Schematic view of the main sea surface current system in the tropical and subtropical North Atlantic (red lines). CC denotes the Canary Current, NEC the North Equatorial Current, NECC the North Equatorial Counter Current, nSEC and sSEC denote the north and south South Equatorial Current respectively and NBC corresponds to the North Brazil Current. The location of the subduction region of the North Atlantic subtropical gyre is also indicated. The climatological sea surface current components, calculated for March for the period 1994–2013 is also shown for reference. These were computed from the Ocean Surface Current Analyses – Real time (OSCAR) data (1° resolution; JPL Physical Oceanography DAAC; developed by ESR). The maps were generated with Matlab 2016a, the M_Map 1.7 toolbox (https://www.eoas.ubc.ca/~rich/map.html) and using the ETOPO2 Global 2 Arc-minute Ocean Depth and Land Elevation (National Geophysical Data Center/NESDIS/NOAA/U.S. Department of Commerce. 2001. ETOPO2, Global 2 Arc-minute Ocean Depth and Land Elevation from the US National Geophysical Data Center (NGDC). Research Data Archive at the National Center for Atmospheric Research, Computational and Information Systems Laboratory. http://dx.doi.org/10.5065/D6668B75. Accessed 1/03/2015).

**Figure 4:**
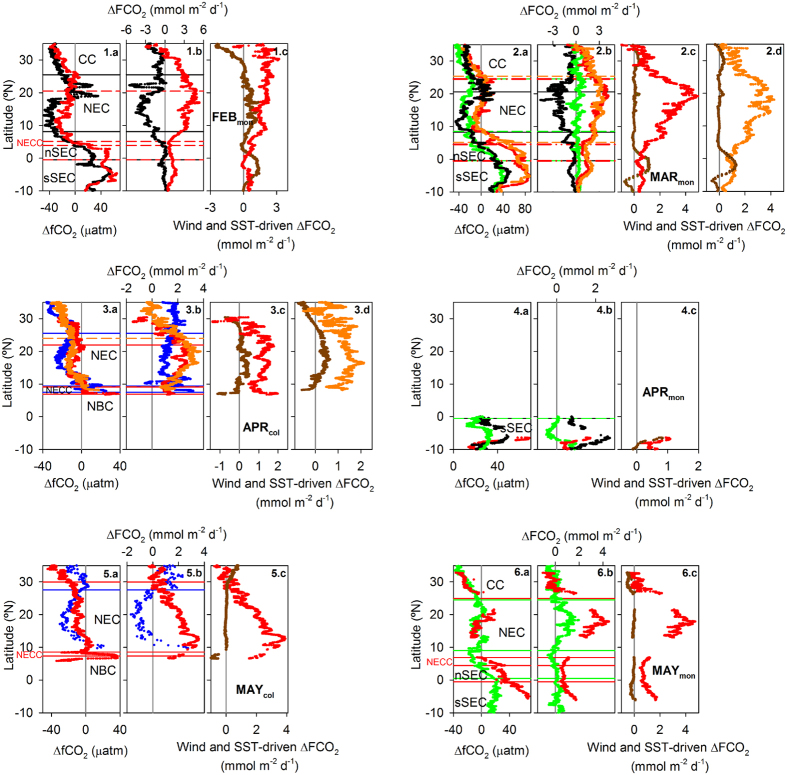
Underway ΔfCO_2_ (shown in panels labeled with a) and CO_2_ flux anomalies (ΔFCO_2_; shown in panels labeled with b). ΔfCO_2_ refers to the difference between sea surface and atmospheric fCO_2_. ΔFCO_2_ was calculated as the difference between the underway CO_2_ flux and the normalized CO_2_ flux. The color code denotes the year of each cruise of the Monte Olivia/Rio Blanco (subscript “mon”) and Colibri (subscript “col”) vessels: 2007 (blue), 2009 (black), 2010 (red and orange) and 2011 (green). The contribution of SST and wind anomalies to ΔFCO_2_ flux during 2010 are also shown in panels labeled with **c** (and d when more than one voyage of each VOS was performed during that same month in 2010): red and orange dots represent the ΔFCO_2_ attributed to SST anomalies, while brown dots are the contribution of wind anomalies to the calculated ΔFCO_2_. The horizontal lines represent the limits of the surface currents identified in this study for reference.

The strongest positive deviations of the underway CO_2_ flux from the normalized CO_2_ flux are almost systematically registered in the cruises performed during 2010, and mainly concentrated in the Northern Hemisphere (Fig. 4, panels labeled with b). SST and wind intensity anomalies are the most significant parameters contributing to the CO_2_ flux anomalies observed in 2010 (Fig. 4 panels labeled with c and d). SST increase during 2010 explains nearly 100% of the CO_2_ flux anomaly during March and May in the Northern Hemisphere (Fig. 4.2c and 4.6.c; red and orange dots). During February and April, negative wind anomalies act together with positive SST anomalies in explaining the calculated positive CO_2_ flux anomalies (Fig. 4.1.c, 4.3.c and 4.3.d; brown dots). Wind intensity determines the gas transfer intensity across the sea-air interface, thus affecting the overall sea-air CO_2_ flux. We observe strong negative surface wind anomalies mainly concentrated in the area of the NEC from February to April 2010 (Fig. 1, e to h). These negative wind anomalies lower the magnitude of the sea-air CO_2_ exchange and feedbacks the impact of increased SST on reducing the CO_2_ absorption capacity in the area of the NEC.

### Basin-scale anomalous sea-air CO_2_ exchange in 2010

SST anomalies in the tropical Atlantic may originate from the zonal or the meridional climate mode. Warming events associated with the tropical Atlantic zonal mode develop under conditions similar to the Pacific El Niño phenomena. Negative wind anomalies in the western equatorial Atlantic precede the reduction of upwelling and the cold tongue in the eastern equatorial Atlantic and overall increased heat content commonly restricted to the equatorial ocean during boreal summer^22^. On the other hand, warming events associated with the Atlantic Meridional Mode (AMM) are characterized by a cross-equatorial SST gradient with positive SST anomalies in the Northern Hemisphere and a Northward shift of the ITCZ, as in boreal spring of 2010 23. The two modes are interconnected through equatorial waves, where boreal spring, tropical Atlantic warming events associated with positive AMM phases can precede boreal summer, equatorial Atlantic Niño events^24^.The intensity of positive AMM phases are linked and exacerbated by the Pacific El Niño events^23^ and positive AMO phases^25^, as observed during 2010^14^. The feedback mechanisms among the AMM, AMO and the El Niño teleconnection mechanisms would thus explain the observed widespread positive SST anomalies in the tropical and subtropical North Atlantic (Fig. 1, a to d). This, in turn, exerts a strong impact over the fCO_2sw_ and sea-air CO_2_ exchange in the tropical and subtropical North Atlantic.

**Figure 5:**
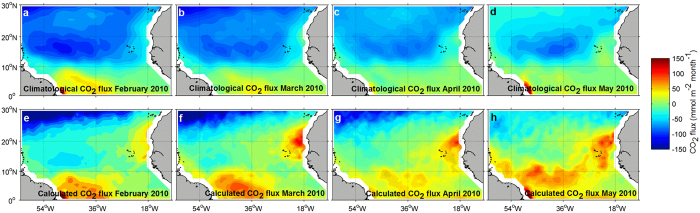
Basin-scale climatological (**a** to **d**) and calculated (**e** to **h**) sea-air CO_2_ flux during February to May 2010. The climatological sea-air CO_2_ flux corresponds to that presented by Landschützer *et al*.^7^ and referenced to the year 2010. Based on this referenced sea-air CO_2_ flux climatology, the anomalous sea-air CO_2_ flux exchange for February to May 2010 was calculated (see discussion). Possitive sea-air CO_2_ flux denotes sea surface CO_2_ outgassing. The maps were generated with Matlab 2016a, the M_Map 1.7 toolbox (https://www.eoas.ubc.ca/~rich/map.html) and using the ETOPO2 Global 2 Arc-minute Ocean Depth and Land Elevation (National Geophysical Data Center/NESDIS/NOAA/U.S. Department of Commerce. 2001. ETOPO2, Global 2 Arc-minute Ocean Depth and Land Elevation from the US National Geophysical Data Center (NGDC). Research Data Archive at the National Center for Atmospheric Research, Computational and Information Systems Laboratory. http://dx.doi.org/10.5065/D6668B75. Accessed 1/03/2015).

According to the sea-air CO_2_ flux climatology calculated from that presented by Landschützer *et al*.^7^ and referenced to the year 2010, the region 0°−30°N 62–10°W is a significant atmospheric CO_2_ sink during boreal spring (from −8.8 Tg C during February to −4.6 Tg C in May; Fig. 5, a to d). The CO_2_ subsaturation of the subtropical North Atlantic largely compensates the net CO_2_ efflux characteristic of the equatorial zone (0.3 Tg C during April to 0.8 Tg C during February, from 10°N to the equator). During the warming event of 2010, the net sea-air CO_2_ exchange for the 0°−30°N 62–10°W region is 1.6 Tg C of CO_2_ outgassing from February to May, contrasting with the high climatological CO_2_ absorption for this area during the same period of time (−29.3 Tg C). The strongest sea-air CO_2_ exchange anomalies compared to the climatology are concentrated in the region of the NEC, from approximately 25 to 10°N. There, the climatological atmospheric CO_2_ uptake of −22.4 Tg C from February to May ceased during 2010 (Fig. 5, e to h). The warm event of this year led to a net CO_2_ emission of 1.2 Tg C for these four months, i.e. a difference in the area of the NEC of 23.6 Tg C.

The impact of the Atlantic warm event of 2010 on the sea-air CO_2_ exchange is not restricted to the area of the NEC. The weakening of the trade winds during this period promoted also a northward shift of the ITCZ, concomitant with a weaker precipitation in the area, which led to an increase of SSS in the equatorial band and enhanced CO_2_ outgassing as shown by Lefèvre *et al*.^14^. Furthermore, the Amazon River presented strong negative discharge anomalies during 2010 which led to a decrease of the atmospheric CO_2_ drawdown associated with its plume^26^. Thus, our estimate of 30.9 Tg C of reduced CO_2_ uptake in the subtropical and tropical Atlantic (0–30°N, 62–10°W) is a very conservative estimation of the impact of the warming event from February to May 2010. Nevertheless, our estimation is already higher than the overall multidecadal sea-air CO_2_ variability reported elsewhere for the entire North Atlantic (20 Tg C)^7^,^8^,^9^. Current surface water warming trends^11^ and the increase in the frequency of extreme climatic events^27^ may threaten the mitigation capacity of the North Atlantic over anthropogenic-driven atmospheric CO_2_ rise and its climatic consequences. In that regard, global ocean reanalysis products could help in further constrain the effects of climate events as that registered in boreal spring of 2010 over the CO_2_ system and the sea-air CO_2_ exchange in the tropical Atlantic.

## Materials and Methods

Two automated CO_2_ instruments based on infrared detection^28^ were installed on board the merchant ships Colibri and Monte Olivia in 2006 and 2008, respectively. The Colibri performs the route Le Havre (mainland France) - Kourou (French Guyana), while the Monte Olivia sails the route Le Havre – Santos (Brazil). In December 2009, the automated CO_2_ equipment installed onboard the Monte Olivia was transferred to the Rio Blanco ship performing the same route. Atmospheric and sea surface fCO_2_ were determined underway. The ships were also equipped with Seabird thermosalinographs and Druck barometers, thus measuring underway SST, SSS and atmospheric pressure (P). In this study, we used the voyages performed by the two ships from February to May 2010, complemented with those performed during the same months in different years for comparison (Supplementary Table S1). Data collected between 35°N and 10°S were used. A total of 16 voyages recorded data in the selected region and for the selected period (Supplementary Table S1). Due to technical problems, the cruise performed during April 2010 by the Rio Blanco only recorded data from 10°S until 6.4°S. Thus, for the remaining cruises performed during April along the Rio Blanco track, only data recorded from 10°S to the equator are used for comparison.

The atmospheric molar fraction of CO_2_ (xCO_2atm_) determined underway the Colibri cruises performed during 2007 and May 2010 were contaminated by the flumes of the ship. Also, during March and April 2011, a problem with the atmospheric pumping onboard the Monte Olivia made xCO_2atm_ unavailable. In these cases, the monthly xCO_2atm_ recorded at the atmospheric stations of Ascension Island (7.97°S, 14.40°W), Maxaranguape (5.51°S, 35.26°W), Ragged Point (13.17°N, 59.43°W), Tenerife (28.31°N, 16.5°W) and Terceira Island (38.77°N, 27.38°W) of the NOAA/ESRL Global Monitoring Division (http://www.esrl.noaa.gov/gmd/ccgg/iadv/) were used. For basin-scale calculations, the monthly xCO_2atm_ recorded at Prospect Hill (Bermuda, 32.30°N, 64.77°W) were also used. Monthly measurements performed at these stations were linearly interpolated at the position where underway measurements were performed. Atmospheric fCO_2_ (fCO_2atm_) was then calculated according to


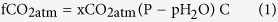


where 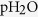 is the water vapor pressure at 100% humidity calculated from SST and SSS and C is the fugacity coefficient calculated from Weiss^29^. The cruise performed by the Colibri during May 2007 lacked P data due to a malfunctioning of the barometer. The monthly atmospheric pressure of the NCEP/NCAR reanalysis project was used in this case. fCO_2atm_ derived from the NOAA/ESRL Global Monitoring Division data was also calculated for the remaining ship tracks used in this study for comparison. Good agreement between the fCO_2atm_ measured onboard and that obtained from the NOAA/ESRL Global Monitoring Division data was verified, with a statistical difference of 0.76 ± 0.02 μatm among both (paired t-test).

Sea-air CO_2_ fluxes (F) were calculated according to


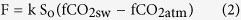


where S_o_ is the solubility of CO_2_^29^ and k is the gas transfer velocity. k was calculated according to^30^:


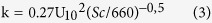


where Sc is the Schmidt number. 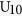 was obtained from the European Centre for Medium-Range Weather Forecasts (ECMWF) monthly reanalysis data set (ERA-interim, 0.25° resolution) and interpolated at the position of the underway measurements.

Basin-scale, monthly SST was calculated from the daily NOAA optimum interpolation (OI) SST V2 data (0.25° resolution; NOAA/OAR/ESRL PSD, Boulder, Colorado, USA; http://www.esrl.noaa.gov/psd/;^31^) and averaged for each month. Monthly sea surface current velocities were obtained from the Ocean Surface Current Analyses – Real time (OSCAR) data (1° resolution; JPL Physical Oceanography DAAC; developed by ESR) and interpolated at each location where measurements were performed.

The global partial pressure of CO_2_ (pCO_2_) climatology presented by Landschützer *et al*.^7^ was used for basin-scale calculations of sea-air CO_2_ fluxes. This climatology is a neural network-based average of extensive data collected from 1998 until 2011. To reference this climatology to the year 2010, we assumed it corresponds to a climatological situation referenced to the year 2004, the median year of the data they used. Thus, based on their climatology, we first calculated fCO_2sw_ from pCO_2_ using a climatological SST (see below). We then added the annual increase in fCO_2sw_ observed in the oligotrophic North Atlantic (1.1 μatm y^−1^)^32^ for the period 2004 to 2010. To obtain an fCO_2atm_ referenced to 2010, the basin-scale interpolation of monthly xCO_2atm_ was used. fCO_2atm_ was then computed using climatological P, SSS and SST. Finally, the climatological sea-air CO_2_ exchange for the basin was calculated using a climatological monthly wind speed (see below). Then, we used the thermodynamic dependence of fCO_2sw_ with SST^16^ to calculate the expected fCO_2sw_ during 2010 from the referenced climatological fCO_2sw_. The sea-air CO_2_ exchange for the anomalous year 2010 was then calculated with the fCO_2atm_, wind fields, atmospheric pressure, SST and SSS observed in 2010.

Monthly wind, atmospheric pressure (NCEP/NCAR reanalysis project) and SST climatologies were computed for the period 1994–2013. Monthly wind and SST anomalies at the basin scale were then computed for February to May 2010. SST anomalies were also computed along the ship tracks by comparing the measured SST with the interpolated, climatological SST. For basin scale S_o_ and pH_2_O calculations, SSS data derived from the Soil Moisture and Ocean Salinity (SMOS) mission and obtained from the Ocean Salinity Expertise Center (CECOS) of the CNES-IFREMER Centre Aval de Traitemenent des Données (CATDS), at IFREMER, Plouzané (France) were used. The available monthly composites (2010 to 2014) were used to calculate the climatological SSS.

### CO_2_ flux normalization and CO_2_ flux anomalies calculation

The different parameters that we considered to potentially contribute to the sea-air CO_2_ flux anomaly of 2010 are SST, SSS, wind intensity, fCO_2atm_ and fCO_2sw_. We interpolated basin-scale climatologies of SST and wind intensity at each position where measurements were taken to obtain the climatological SST and wind intensity for each cruise. We also calculated latitudinal averages of SSS and fCO_2atm_ of monthly cruises of each VOS. Then, we used the thermodynamic dependence of fCO_2sw_ with SSS and SST^16^ to calculate the theoretical fCO_2sw_ at these averaged SSS and climatological SST, and thus calculate a normalized CO_2_ flux for each cruise together with averaged fCO_2atm_ and climatological wind speed. The CO_2_ flux anomaly (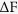) was then calculated as the difference between the calculated underway CO_2_ flux and the normalized CO_2_ flux.

To elucidate the relative contribution of the different physical parameters to the CO_2_ flux anomaly observed during February to May 2010, we followed the expression presented by Lefèvre *et al*.^14^:


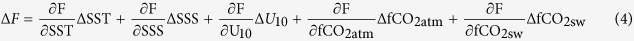


where U_10_ is the wind intensity at 10 m above sea level.

Throughout this study, the errors associated with our results correspond to the standard error of the estimates.

## Additional Information

**How to cite this article**: P. Ibánhez, J. S. *et al*. Collapse of the tropical and subtropical North Atlantic CO_2_ sink in boreal spring of 2010. *Sci. Rep.* **7**, 41694; doi: 10.1038/srep41694 (2017).

**Publisher's note:** Springer Nature remains neutral with regard to jurisdictional claims in published maps and institutional affiliations.

## Supplementary Material

Supplementary Material
