# Supplementary material for: Collapse of the tropical and subtropical North Atlantic CO2 sink in boreal spring of 2010
Source: Sci Rep. 2017 Jan 30;7:41694. doi: 10.1038/srep41694 (PMC5278357; doi:10.1038/srep41694)

Supplementary material of manuscript:

Collapse of the tropical and subtropical North Atlantic CO_2 sink in boreal spring of 2010

J. Severino P. Ibánhez[1*], Manuel Flores[1], Nathalie Lefèvre[2]

1. Department of Oceanography – DOCEAN, Federal University of Pernambuco – UFPE, Av. Arquitetura, s/n, Cidade Universitária, 50740-550, Recife-PE, Brazil

2. IRD-LOCEAN, Sorbonne Universités (Université Pierre et Marie Curie-CNRS-MNHN), 4 place Jussieu, 75252 Paris Cedex 05, France

*Corresponding author: J.S.P. IBÁNHEZ

e-mail: pinoibaj@tcd.ie

List of Supplementary Materials

Supplementary text

Supplementary Table S1

Supplementary Figure S1

Supplementary Figure S2

Supplementary Text

Surface currents discrimination along the VOS tracks

The CC is the eastern boundary current of the subtropical North Atlantic gyre, which transports waters from the North Atlantic to the tropical band, feeding the NEC [1]. Further South, the system of currents spread mainly in the zonal direction, with the westward NEC and SEC as the equatorial limits of the North and South Atlantic gyres respectively, separated by the NECC and the Equatorial Undercurrent which flow eastwards. The SEC is the main transport pathway of surface Southern Hemisphere waters into the Northern Hemisphere and presents different branches (the North and South SEC; nSEC and sSEC) [2]. When arriving close to the coast of Brazil, the SEC splits into the Brazil Current (BC) which flows southward, and the NBC flowing along the Northern coast of Brazil.

Monthly zonal sea surface current velocities (cm s^{-1}) corresponding to the month each cruise was performed were downloaded from the Ocean Surface Current Analyses – Real time (OSCAR) data, obtained from JPL Physical Oceanography DAAC and developed by ESR (1/3º resolution). The OSCAR data was interpolated at the position where underway measurements were performed to estimate the limits of the surface currents crossed by the VOS.

The latitudinal limit between the CC (southward propagation) and the NEC (westward propagation) is established at the latitude where the latitudinal component of the velocity equals zero and/or when the westward component of the velocity intensifies. In the case of the Colibri, the latitudinal limit between the subtropical gyre and the NEC is stablished at the latitude where the westward component of the velocity intensifies. The southern limit of the NEC was stablished where the zonal component of the velocity changes to eastward propagation when the NECC is present, or until an intensification of the westward propagation caused by the SEC

(Monte Olivia/Rio Blanco) or the NBC (Colibri) when not present. Finally, two branches of the SEC were observed in the interpolated surface velocities along the Monte Olivia/Rio Blanco tracks, hereby denoted North SEC (nSEC) and South SEC (sSEC). The two branches of the SEC appeared separated by an area of weakened zonal velocity or even reversal of direction around the equator, and contrasting latitudinal components (Supplementary Figure 1). While the nSEC shows a northward component (thus feeding the NBC), the sSEC propagates to the South, feeding the BC (included in the sSEC in this latitudinal discrimination of currents). In this discrimination of the data as a function of the sea surface current system, the data identified as affected by the upwelling in the CC and that affected by the Amazon plume (SSS<35 psu in the Colibri voyages) were removed to avoid misinterpretations caused by these phenomena.

References

1. Nieto, K., Demarcq, H. & McClatchie, S. Mesoscale frontal structures in the Canary Upwelling System: New front and filament detection algorithms applied to spatial and temporal patterns. *Remote Sensing of Environment* **123,** 339–346 (2012).
2. Mercier, H., Arhan, M. & Lutjeharms, J. R. E. Upper-layer circulation in the eastern Equatorial and South Atlantic Ocean in January–March 1995. *Deep Sea Research Part I: Oceanographic Research Papers* **50,** 863–887 (2003).

Supplementary Table S1. Voyages of the two VOS lines (France-French Guyana and France-Brazil) used in this study. The limits of the ship tracks used here and the dates the ship performed each track are also shown.

Dates of the voyages	Vessel name	Minimum latitude	Maximum latitude	Route	Representative month
9-14 February 2010	Rio Blanco	10ºS	35ºN	Brazil-France	February
8-14 February 2009	Monte Olivia	10ºS	35ºN	France-Brazil	February
2-8 March 2009	Monte Olivia	10ºS	35ºN	Brazil-France	March
28 February – 7 March 2010	Rio Blanco	10ºS	35ºN	France-Brazil	March
23-28 March 2010	Rio Blanco	10ºS	35ºN	Brazil-France	March
21-28 March 2011	Rio Blanco	10ºS	35ºN	France-Brazil	March
14 April 2009	Monte Olivia	10ºS	equator	Brazil-France	April
20 April 2010	Rio Blanco	10ºS	equator	France-Brazil	April
23 April 2011	Rio Blanco	10ºS	6°S	Brazil-France	April
9-15 May 2010	Rio Blanco	10ºS	33ºN	Brazil-France	May
9-16 May 2011	Rio Blanco	10ºS	35ºN	France-Brazil	May
1-5 April 2010	Colibri	7ºN	30ºN	France-French Guyana	April
21-30 April 2007	Colibri	6ºN	35ºN	France-French Guyana	April
11-19 April 2010	Colibri	8ºN	35ºN	French Guyana- France	April
17-23 May 2010	Colibri	6ºN	35ºN	France-French Guyana	May
7-14 May 2007	Colibri	9ºN	35ºN	French Guyana- France	May

Supplementary Figure S1. Interpolated sea surface current components and underway SST anomalies. Monthly surface water current components (zonal, u and latitudinal, v; positive u denotes eastward propagation, while positive v denotes northward propagation) obtained from the OSCAR data and interpolated at the position where underway measurements were performed are shown. Underway SST anomalies, calculated from underway SST and a Reynolds' SST OI climatology are also shown. Horizontal lines represent the limits of each surface current. Each color represents a year (blue 2007, black 2009, red and orange 2010 and green 2011) and subscript "mon" and "col" denotes data collected onboard the Monte Olivia/Rio Blanco and Colibri, respectively.

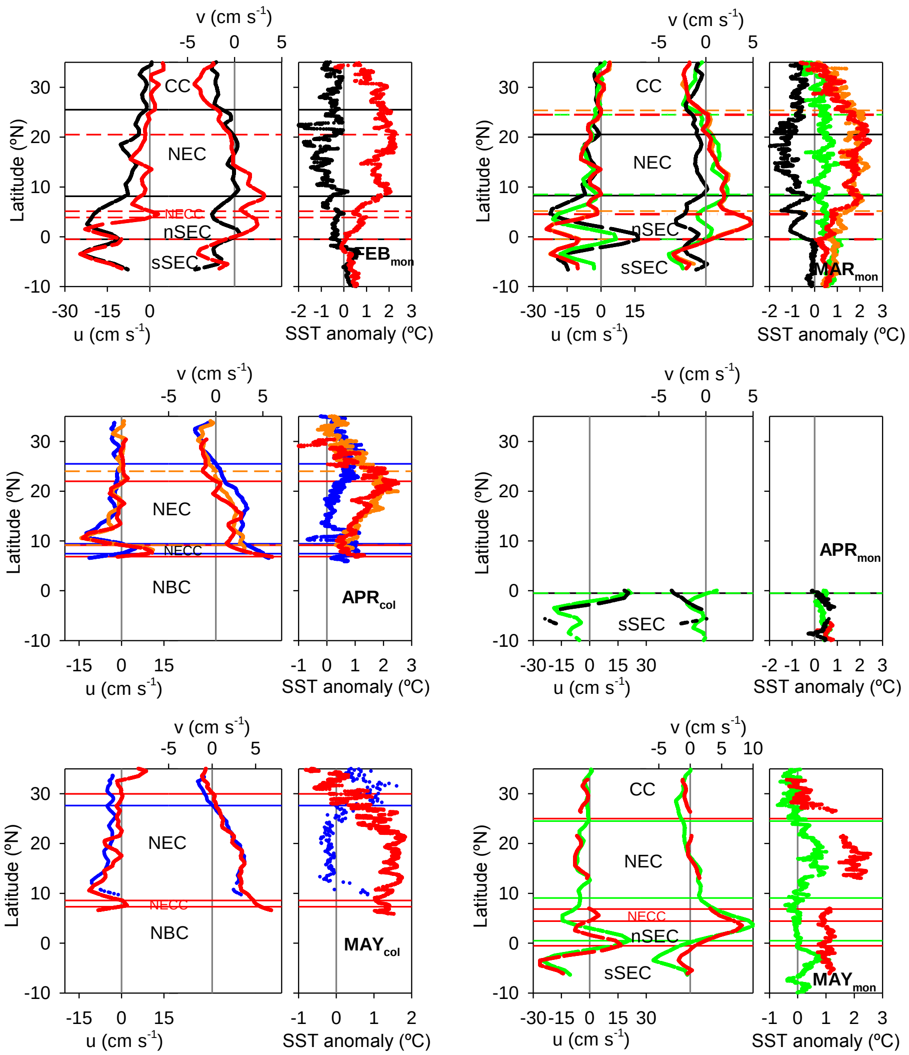
v (cm s-1)
Latitude (ºN)
u (cm s-1)
SST anomaly (ºC)
CC
NEC
NECC
nSEC
sSEC
NBC
FEBmon
MARmon
APRcol
APRmon
MAYcol
MAYmon

Supplementary Figure S2. Underway and normalized CO_2 flux for the cruises used in this study. Normalized CO_2 fluxes were calculated from climatological SST and wind and latitudinal averages of SSS and fCO_{2atm} of monthly cruises of each VOS. The color code used represents the year of each cruise: blue represents 2007, black 2009, red (and orange when more than one cruise was performed during the same month) 2010, and green 2011. Subscript "mon" denotes data collected on the voyages of the Monte Olivia/Rio Blanco, while subscript "col" denotes data collected on the voyages of the Colibri.

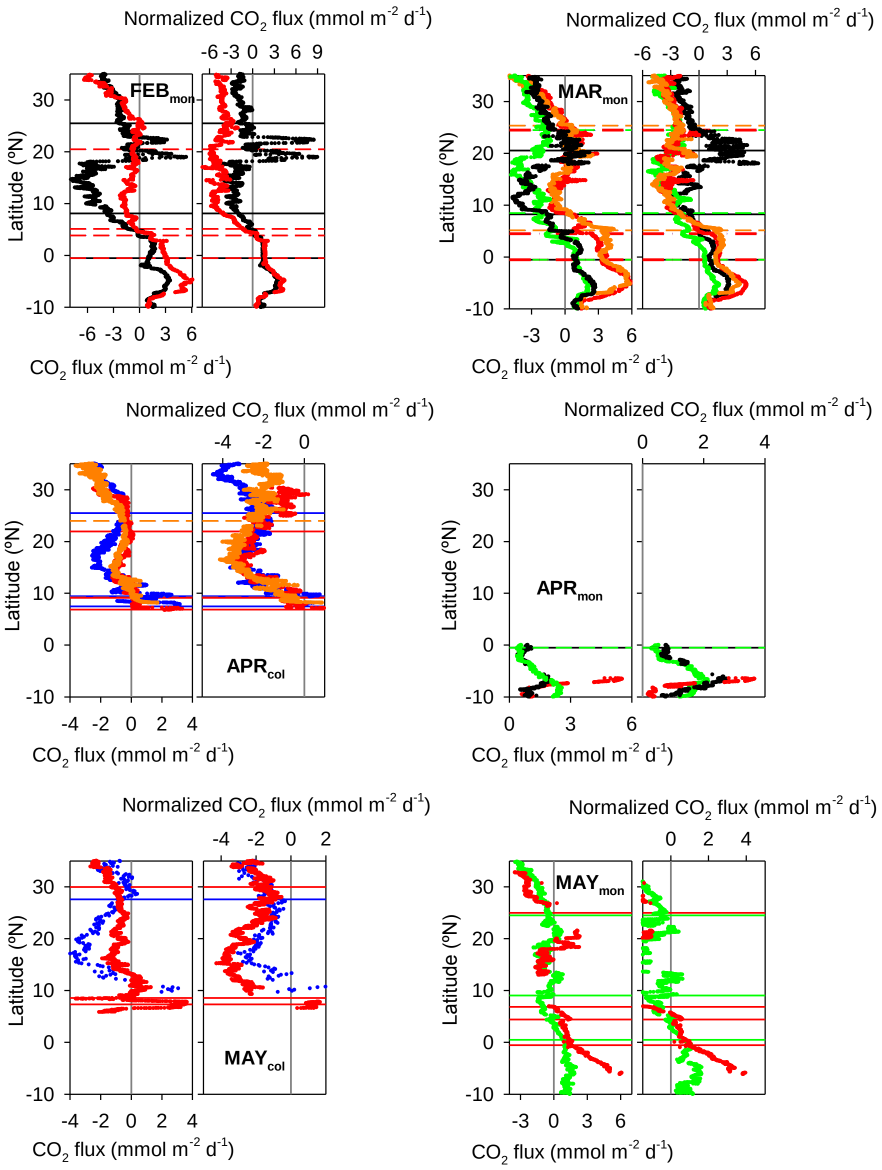

Supplement: Supplementary Material [file srep41694-s1.pdf]
